# Effect of Bee Pollen Addition on the Polyphenol Content, Antioxidant Activity, and Quality Parameters of Honey

**DOI:** 10.3390/antiox10050810

**Published:** 2021-05-20

**Authors:** Celina Habryka, Robert Socha, Lesław Juszczak

**Affiliations:** Department of Food Analysis and Evaluation of Food Quality, Faculty of Food Technology, University of Agriculture in Krakow, Balicka 122 Str., 30-149 Krakow, Poland; celina.habryka@gmail.com (C.H.); robert.socha@urk.edu.pl (R.S.)

**Keywords:** honey, bee pollen, phenolic compounds, antioxidant activity

## Abstract

Bee pollen is regarded as a valuable source of bioactive substances. Honey enrichment with bee pollen seems to be the most popular way to introduce this bee product into a human diet. The aim of this study was to determine the influence of the addition of bee pollen to honey on the content of selected biologically active pollen components, antioxidant activity, and quality parameters, as well as sensory properties. On the basis of the obtained results, it was established that enriching honey with bee pollen resulted in a significant increase in the level of phenolics, including both flavonoids and phenolic acids, of which kaempferol and gallic acid were present in the highest level. As a result of increasing addition of bee pollen, an increase in the antioxidative, antiradical, and reducing activities of honey was observed. However, the addition of bee pollen to honey resulted in the deterioration of its sensory properties. A decrease in brightness, clarity, and uniformity of color, as well as a decrease in the perception of fragrance, was observed. In the assessment of texture, a decrease in smoothness and spread ability in the mouth was observed, with a significant increase in the feeling of sandiness. In contrast, the evaluation of taste revealed a marked increase in sharpness, acid taste, bitterness, and persistence of the aftertaste, with a simultaneous decrease in sweetness. Honey enrichment with bee pollen resulted in a significant increase in the content of water-insoluble substances, free acidity, specific conductivity, and proline content, with a slight decrease in the content of glucose and fructose.

## 1. Introduction

Bee pollen is formed in the anther cells of plants. While collecting it, honey bees mix the pollen with saliva and/or nectar and transport it to the hive in loads. Bee pollen forms loads of a characteristic color for individual plants, depending on the flowers visited by the bees [1]. Its chemical profile varies significantly and depends on its origin and weather conditions during anther forming and maturing [1,2].

Bee pollen is regarded as a product rich in bioactive ingredients. The presence of over 250 compounds has been reported in this bee product [2,3,4]. The main groups of biologically active bee pollen components include proteins, carbohydrates, lipids, phenolic compounds, bioelements, and vitamins [3,5]. The total protein content is over 20%, and the main proteins found in bee pollen are albumins, globulins, glutelins, prolamines, and enzymes. Bee pollen contains all the essential amino acids needed for the human organism [6]. Essential fatty acids (EFA) constitute the highest amount of lipids found in bee pollen. Among the essential fatty acids, γ-linolenic, linoleic, and arachidonic acids are most abundant [1,3]. The phenolic compounds found in bee pollen include flavonoids, including catechins and leucoanthocyanidins, and phenolic acids [7]. The content of flavonoids in bee pollen can be as high as 2.5%, and they occur mainly as glycosides [3]. Bee pollen loads also contain kaempferol derivatives, apigenin, luteolin, and the derivatives of quercetin, the latter mainly present as rutin (quercetin-3-*O*-rutinoside). The most common phenolic acids found in bee pollen include *p*-coumaric, chlorogenic, and ferulic acids and their derivatives [3,4,7]. Bee pollen is also a product rich in carotenoids and vitamins, including tocopherols and, in smaller quantities, calciferol. Furthermore, bee pollen is a rich source of valuable macro- and microelements for the human body [1,8].

The biologically active compounds present in bee pollen include substances with various properties, e.g., phytosterols, organic acids, and enzymes. The compounds possessing antibacterial properties include inhibins and phenolic acids, triterpenes, and phytohormones [7,9]. Bee pollen is a valuable source of vitamin E, which, due to its antioxidant properties, protects unsaturated fatty acids and some vitamins against oxidation. Bee pollen also contains quercetin, an antioxidant that reduces the LDL cholesterol content in the human body and also has anti-atherosclerotic properties [9]. Polyphenols play an important role in detoxifying the human body after drug or alcohol poisoning. They inhibit the activity of the enzymes that are responsible for the formation of inflammation. They have also an antibiotic effect on yeast fungi and bacteria pathogenic for humans. Bee pollen has an anti-atherosclerotic effect because it reduces the content of total lipids, total cholesterol, and triacylglycerides in the blood serum and also reduces the ability to aggregate platelets. Bee pollen also exhibits a high nutritional value and supplements deficiencies in vitamins, bioelements, and exogenous amino acids [3,4,9].

Due to its antibacterial activity, bee pollen is effective in the treatment of ulcerative colitis, constipation, and diarrhea. The enzymes contained in this bee product enhance the secretory activity of the stomach and ensure proper intestinal peristalsis. Bee pollen produces a protective impact on the liver and supports the reconstruction of liver tissue. It also has a detoxifying effect and normalizes disturbed protein metabolism [6,9]. Bee pollen helps to improve the performance of the heart and thus reduce the risk of heart failure. It exerts a positive effect on the hematopoietic system; therefore, it can be used in anemia, in particular in iron deficiency anemia [3,9]. It also protects against ischemic heart disease and strokes. The extracts of bee pollen increase insulin secretion, while bee pollen consumed by diabetics effectively helps to reduce their blood sugar levels [9].

Bee products, including bee pollen, are gaining increasing recognition among consumers due to the presence of bioactive compounds. Honey enrichment with bee pollen seems to be the most popular way to introduce this bee product into a human diet. The aim of this study was to estimate the effect of enriching multiflower honey with bee pollen on the phenolic profile, antioxidant properties, sensory characteristics, and parameters characterizing its commercial quality.

## 2. Materials and Methods

### 2.1. Materials

The materials used were multiflower honey (District Beekeeping Cooperative “Pszczelarz,” Krakow, Poland) and micronized bee pollen (Biopharmaceutical Laboratory Arria, Krakow, Poland). On the basis of preliminary sensory evaluation, the honey samples were supplemented with bee pollen in the amounts of 5%, 10%, 15%, 20%, and 25%.

### 2.2. Methods

#### 2.2.1. Total Phenolic, Flavonoid, Phenolic Acid, Anthocyanin, and Carotenoid Content

Determination of the antioxidant potential of multiflower honey samples and the samples enriched with bee pollen was made using an ethanolic–water extract with an initial concentration equal to 0.2 g/mL. An appropriate amount of the sample was dissolved in ethanol:water solution (1:1 *v*/*v*), centrifuged (3000× *g*; 15 min), and filtered through filter paper. The analysis was conducted on a UV–Vis V-630 spectrophotometer (Jasco, Tokyo, Japan).

The total phenolic content (TPC) was measured using the Folin–Ciocalteu method following the procedure developed by Singleton and Rossi [10], and the results were expressed as milligrams gallic acid equivalents (GAE)/100 g of sample. The total flavonoid content (TFC) was determined in the reaction with aluminum chloride based on the procedure described by Ardestani and Yazdanparast [11], and the results were expressed as milligrams quercetin equivalents (QE)/100 g of sample. The total phenolic acid content (TPAC) was measured using the reaction of the sample with Arnov’s reagent according to the procedure developed by Szaufer-Hajdrych [12], and the results were expressed as milligrams caffeic acid equivalents (CAE)/100 g of sample. The total anthocyanin content (TAC) was estimated using the procedure developed by Rababah et al. [13], and results were expressed as milligrams cyanidin-3-glucoside equivalents (CGE)/100 g of sample. The total carotenoid content (TCC) was determined by the method developed by Boussaid et al. [14], and the results were expressed as milligrams β-carotene equivalents/100 g of sample.

#### 2.2.2. Phenolic Profile Determination by HPLC-DAD

The phenolic composition was determined using high-performance liquid chromatography (HPLC). Polyphenolic compounds were extracted using ethyl acetate according to the procedure developed by Socha et al. [15]. Sample solutions (of 10 g/50 mL) were acidified with HCl solution to reach pH 2 and then saturated with sodium chloride (3 g/10 mL). The obtained solutions were extracted with three portions of ethyl acetate. The obtained extracts were combined, and ethyl acetate was evaporated at 40 °C under argon. The residue was dissolved in 5 mL of methanol and purified by using Millex-LCR syringe filters. Qualitative and quantitative analyses of identified phenolic acids and flavonoids were performed using a high-performance liquid chromatograph (HPLC LC-Net II/ADC; Jasco, Japan) equipped with a diode array detector (DAD) (MD-2018 Plus; Jasco, Japan) [16]. The analyses were conducted on an RP-18 column (Purospher: 250 mm × 4 mm, 5 µm; Merck, Darmstadt, Germany) at the flow rate of 1 mL/min and at 30 °C. The eluent system constituted a linear gradient with the two different phases: phase A was a water solution of acetic acid (2.5 g/100 mL, *v*/*v*), and phase B was acetonitrile. The elution was performed as follows: For the first 10 min of analysis, a linear gradient was applied with a phase B contribution that increased from 3% to 8%, followed by an increase in the phase B contribution to 15%, 20%, 30%, and 40% at 20, 30, 40, and 50 min, respectively. The qualitative analysis of both phenolic acids and flavonoids was conducted by comparison of the retention times of analyzed phenolic acids with standards as well as by comparison of the UV spectra of the analyzed phenolic compounds with those obtained for the standards of phenolics by using a DAD detector. In contrast, the quantitative analysis of the phenolic compounds under study was conducted on the basis of the calibration curves that were plotted separately for each standard in a concentration range from 0.02 to 0.2 mg/mL.

#### 2.2.3. Antiradical Properties and Reducing Power

Evaluation of the total antioxidant activity was performed following the procedure developed by Prieto et al. [17], and the obtained results were expressed as equivalents of ascorbic acid (mmol AAE/100 g). Assessment of the antiradical activity against DPPH radicals (Sigma-Aldrich, Hamburg, Germany) was performed according to the procedure developed by Blois et al. [18], and the obtained results were expressed as equivalents of Trolox in millimoles per 100 g of sample (mmol TE/100 g). Evaluation of the antiradical activity against ABTS cations radicals (Sigma-Aldrich, Germany) was performed following the method developed by Baltrušaityte et al. [19], and the obtained results were expressed as equivalents of Trolox in millimoles per 100 g of sample (mmol TE/100 g). The ferric reducing antioxidant power was determined using the FRAP method following the procedure developed by Benzie et al. [20], and the obtained results were expressed as micromoles of Fe(II) ions per 100 g of sample (µmol Fe(II)/100 g). The cupric reducing ability was determined by the CUPRAC method using the reaction of the sample with neocuproine (Sigma-Aldrich, Germany) following the method developed by Apak et al. [21], and the results were expressed as micromoles of Trolox per 100 g of sample (µmol TE/100 g).

#### 2.2.4. Sensory Properties

Sensory analysis of the honey enriched with bee pollen was performed on the basis of an evaluation by a 14-person sensory panel. The sensory analysis was performed by the quantitative description method according to the PN-EN ISO 13299:2016 standard [22]. The used research methodology included evaluation of the color, smell, texture, and taste of the multiflower honey and the honey samples supplemented with bee pollen. With respect to the color determination, the following descriptors were assessed: brightness, clarity, cloudiness, and uniformity. The descriptors specifying taste included sweetness, acid taste, bitterness, sharpness, foreign taste, and aftertaste (persistence). Among sensory descriptors used for the smell assessment, the following were assessed: honey-like, molasses, floral, sweet, waxy, and foreign smell. The texture of the samples was evaluated taking into account the following parameters: smoothness, adhesiveness, meltability, and sandiness. The intensity of perception was rated on the following scale: 0 (imperceptible), 1 (barely perceptible), 2 (hardly perceptible), 3 (moderately perceptible), (strongly perceptible), and 5 (very strongly perceptible).

To estimate the acceptance of the honey samples, a seven-point hedonic scale was used. Color, texture, smell, and taste were evaluated. The obtained scores were rated in the form of a scale from 0 (I dislike very much) to 7 (I like very much) [23].

#### 2.2.5. Quality Parameters

The qualitative parameters of multiflower honey and the honey samples supplemented with bee pollen were analyzed in accordance with the requirements of the Ministry of Agriculture and Rural Affairs in Poland, 2009 [24]. The content of water-insoluble matter was determined by the gravimetric method. Free acidity was analyzed by the titration method using a TitroLine easy titrator (Schott, Mainz, Germany). Specific conductivity was determined using a CPC 501 conductometer (Elmetron, Zabrze, Poland). Determination of the saccharide content (i.e., glucose, fructose, and saccharose) was conducted by high-performance liquid chromatography using refractometric detection (HPLC-RI) (HPLC-LaChrom D-7000; Merck-Hitachi, Tokyo, Japan). Separation of saccharides was carried out using a Purospher Star NH_2_ column (250 × 4 mm, 5 μm) and thermostatted at 30 °C. A mixture of acetonitrile:water (80:20, *v*/*v*) with a flow rate of 1 mL/min was used as a mobile phase. Determination of the 5-hydroxymethyfurfural (HMF) content was made by high-performance liquid chromatography with UV detection at *λ* = 285 nm (HPLC-UV) (HPLC-LaChrom D-7000; Merck-Hitachi, Japan). Determination of the HMF content was carried out using an Eclipse XDB-C18 column (250 × 4.6 mm, 5 μm). A mixture of water:methanol (90:10, *v*/*v*) with a flow rate of 1 mL/min was used as a mobile phase.

#### 2.2.6. Statistical Analyses

The obtained data were expressed as the means of three repetitions and standard deviation (mean ± SD). To calculate differences between mean values, the data were treated by one-way analysis of variance, and the least significant difference (LSD) between the mean values was calculated using the Fisher LSD test at a level of significance of *p* < 0.05. In addition, the coefficients of the linear correlations between the selected variables were calculated, and their significance was verified at the level of 0.05. All calculations were performed using the statistical software package Statistica 11.0 (StatSoft Inc., Tulsa, OK, USA).

## 3. Results and Discussion

### 3.1. The Impact of Honey Enrichment with Bee Pollen on the Total Content of Polyphenolic Compounds

Table 1 shows the results of evaluation of the total content of polyphenolic compounds, flavonoids, phenolic acids, anthocyanins, and carotenoids in the mulitflower honey and in the samples supplemented with bee pollen. In the case of the analyzed multiflower honey, the total phenolic content was at the level of ca. 30 mg GAE/100 g (Table 1), and this value was similar to that reported by Tomczyk et al. [25]. On the contrary, Halagarda et al. [26] observed a lower content of polyphenols in Polish multiflower honeys. With the increasing addition of bee pollen, the phenolic content in the analyzed honeys also increased, reaching a value of 178.26 mg GAE/100 g for the highest level of bee pollen addition (Table 1).

Majewska and Trzanek [27] determined a similar result, close to the results obtained for the level of polyphenols, of 83.3 mg GAE/100 g of polyphenols in honey with 10% addition of bee pollen. In the samples of commercial honeys enriched with bee pollen, the determined values of the phenolic content varied in the range from 70.88 to 119.94 mg GAE/100 g [28]. This significant increase in the total content of polyphenolic compounds in honeys enriched with bee pollen indicates that the used additive is a rich source of them. According to various data, the total phenolic content in bee pollen can reach 2132 mg GAE/100 g [1], 3215 mg GAE/100 g [7], or even 4300 mg GAE/100 g [8]. However, the total phenolic content in bee pollen significantly depends on both the type and the origin of plant sources from which it was obtained [5]. According to the quoted authors, the total phenolic content can vary from 420 mg GAE/100 g in bee pollen obtained from magnolia (*Magnolia*) flowers to 2960 mg GAE/100 g in bee pollen from white nettle (*Lamium*) flowers. In contrast, LaBlanc et al. [29] established the phenolic content at a level of 3485 mg GAE/100 g in bee pollen obtained from mimosa (*Mimosa*) flowers. Flavonoids, classified as natural antioxidants, are an important group of biologically active compounds present in plants. They penetrate honey, together with bee pollen and propolis; therefore, their content in honey depends on the presence of small quantities of these bee products in honey. In the multiflower honey, the total content of flavonoids was determined as 2.77 mg QE/100 g. The obtained results were slightly lower when compared to values reported in the literature for Polish multiflower honeys [30,31]. The honey samples enriched with bee pollen were characterized by a significant increase in the total flavonoid content (Table 1). Already a 5% addition of bee pollen doubled the content of flavonoids, and 25% addition led to an increase in their content to 16.39 mg QE/100 g. Juszczak et al. [28] established the flavonoid content in commercial honeys enriched with bee pollen within a range of 8.52–12.92 mg QE/100 g. The literature data show that the flavonoid content in bee pollen can reach up to 1349 mg QE/100 g [29], or even up to 2843 mg QE/100 g [1], and the main representatives of this group of compounds are quercetin, kaempferol, and their derivatives [3,4]. Thus, this confirms the hypothesis that the addition of bee pollen to honey leads to a significant increase in the flavonoid content.

In addition to flavonoids, phenolic acids are another important and characteristic group of polyphenols present in honey. In the analyzed multiflower honey, the total phenolic acid content was determined to be 11.02 mg CAE/100 g (Table 1). The honey samples supplemented with bee pollen exhibited an increase in the total phenolic acid content in a range from 16.65 to 24.44 mg CAE/100 g for 5% and 25% addition of bee pollen, respectively. According to Kocot et al. [4], the phenolic acid content fond in bee pollen can reach even 190 mg/100 g, with gallic acid being their most important representative. In the analyzed honey, the anthocyanin content amounted to 2.01 mg/100 g (Table 1), which fell within a relatively narrow range reported in the literature [13,32]. The increasing addition of bee pollen to honey resulted in an increase in the total anthocyanin content. With 25% addition of bee pollen, the level of this group of compounds reached 11.32 mg/100 g (Table 1). The observed increase in the total anthocyanin content confirms that bee pollen contains large quantities of these compounds [1]. In addition to anthocyanins, honey also contains carotenoids, which demonstrate antioxidant properties. The content of this group of coloring agents in the multiflower honey was much lower when compared with the anthocyanin content and was at the level of 0.138 mg/100 g (Table 1), confirming earlier literature data [14,33]. A higher level of these compounds was reported by Alqarni et al. [32]. The addition of bee pollen to honey led to an increase in the carotenoid content, ranging from 0.311 to 2.333 mg/100 g (Table 1), and this confirms the hypothesis about the high content of these compounds being responsible, among others, for the bee pollen color.

As the literature data imply, a significant positive linear correlation was observed between the total polyphenol content and the content of individual groups of compounds [13,16,30,33]. In addition, during these studies, a significant linear correlation between the total content of phenolic compounds and the total content of flavonoids (r = 0.9992), phenolic acids (r = 0.9714), anthocyanins (r = 0.9925), and carotenoids (r = 0.9837) was observed.

### 3.2. The Impact of Bee Pollen Addition on the Polyphenolic Content

Flavonoids and phenolic acids represent important groups of antioxidants found in honey and bee products. In mulitflower honey, the most common flavonoids are quercetin, kaempferol, chrysin, and naringenin [26,34]. In this study, four flavonoids and six phenolic acids were identified and quantified, and the content of individual phenolic compounds is presented in Table 2. In the case of multiflower honey, kaempferol was the predominant flavonoid, and with the increasing addition of bee pollen to honey, the concentration of this flavonoid increased over 40 times, reaching a level of 2.183 mg/100 g (Table 2), and this implies that bee pollen has a high content of this compound.

The increasing addition of bee pollen to honey also influenced the increase in the quercetin content, ranging from 0.237 to 0.731 mg/100 g for samples with 5% and 25% addition of bee pollen, respectively (Table 2). According to Kieliszek et al. [3] and Kocot et al. [4], kaempferol and quercetin are predominant flavonoids in bee pollen. With the increase in bee pollen addition to honey, the levels of two other flavonoids, galangin and chrysin, also rose (Table 2). Already a 5% addition of bee pollen doubled the galangin content, and at its maximum levels of 25%, the content of this flavonoid reached 0.17 mg/100 g (Table 2), while with the maximum addition of bee pollen, the chrysin content amounted to 0.054 mg/100 g (Table 2). Similar to flavonoids, phenolic acids present in honeys and bee products are also an important and characteristic group of phenolic compounds. The obtained results concerning the contents of individual phenolic acids found as their free forms in the analyzed multiflower honey and in honey samples supplemented with bee pollen are provided in Table 2. Gallic acid turned out to be the predominant one among all the phenolic acids identified in the investigated multiflower honey. Its level amounted to 0.217 mg/100 g of the honey (Table 2). According to Socha et al. [30,31], gallic acid is found to be a predominant phenolic acid in Polish multiflower honeys. The increasing level of bee pollen in honey resulted in an increase in the gallic acid content, ranging from 0.526 to 1.494 mg/100 g for samples with 5% and 25% (maximum) addition of bee pollen, respectively (Table 2). *p*-Coumaric acid was determined to be 0.136 mg/100 g in the multiflower honey (Table 2), and this fact confirms previous literature reports [30,31]. The *p*-coumaric acid content in honey samples supplemented with bee pollen increased with the increase in the additive. The content of *p*-coumaric acid amounted to 0.296 and 0.485 mg/100 g for 5% and 25% bee pollen levels, respectively (Table 2). Thakur and Nanda [1] reported that bee pollen is a good natural source of *p*-coumaric acid. The content of ferulic acid in the multiflower honey was established at a level of 0.095 mg/100 g (Table 2). The presence of this acid in Polish honeys was earlier established by Socha et al. [31,34]. The enrichment of multiflower honey with the increasing addition of bee pollen led to an increase in the ferulic acid content, ranging from 0.191 to as high as 0.503 mg/100 g for 5% and 25% addition of bee pollen, respectively (Table 2). This indicates that bee pollen is also a source of this phenolic acid, as well as confirming its presence in this bee product [1]. In the analyzed multiflower honey, the protocatechuic acid content was established at a level of 0.070 mg/100 g. The content of this acid significantly increased with the addition of bee pollen, reaching a level of 0.535 mg/100 g at the highest, 25%, addition of bee pollen. Another compound found in the studied multiflower honey was *p*-hydroxybenzoic acid (Table 2), and increasing bee pollen addition to honey contributed to an increase in this acid’s content to the level of 0.133 mg/100 g with 25% addition of bee pollen. The presence of *p*-hydroxybenzoic acid in bee pollen was already previously confirmed by Thakur and Nanda [1]. Caffeic acid was another phenolic acid identified and determined in the studied multiflower honey, and its level was the lowest among the phenolic acids tested. Its content amounted to 0.026 mg/100 g (Table 2). According to Socha et al. [30,31,34], the content of this acid in Polish multiflower honeys ranges from trace amounts to 0.050 mg/100 g. With the increasing addition of bee pollen, the caffeic acid content in the honey increased from 0.034 to 0.163 mg/100 g.

### 3.3. The Impact of Bee Pollen Addition on the Antioxidant Activity

The multiflower honey was characterized by the total antioxidant activity, which amounted to 9.24 mmol AAE/100 g (Table 3). The increasing addition of bee pollen led to an increase in the antioxidant activity of honey from 9.90 to 12.41 mmol AAE/100 g (Table 3) due to the replacement of a part of the honey with bee pollen, which is characterized by a much higher antioxidant potential than honey [7,9].

The antiradical activity is determined as the ability to neutralize free radicals. The results of antiradical activity determination in the multiflower honey and in the samples enriched with bee pollen are shown in Table 3. Antiradical activity measured in the reaction of ABTS cation radicals with the multiflower honey was established at a level of 1.78 mmol TE/100 g. With the increased addition of bee pollen to honey, the antiradical activity increased from 5.40 to 13.48 mmol TE/100 g for 25% bee pollen content (Table 3). Juszczak et al. [28] found an increase in antiradical activity in commercial honeys enriched with bee pollen. The antiradical activity of the multiflower honey and the samples enriched with bee pollen was also measured in the reaction with DPPH radicals (Table 3). Similar to ABTS cations, in this case also, the increasing addition of bee pollen to honey resulted in an increase in antiradical activity to the level of 2.22 mmol TE/100 g for the highest bee pollen addition (Table 3). This fact confirms the previous observations for samples of commercial honeys enriched with bee pollen [28]. The reducing capacity of the multiflower honey was established at a level of 233.9 μmol Fe(II)/100 g (Table 3). The obtained result was within the extensive range of values observed in Polish multiflower honeys [28,35]. The addition of bee pollen to honey resulted in a significant rise in the reducing capacity of the samples. Already a 5% level of bee pollen increased the reducing capacity to the level of 497.0 μmol Fe(II)/100 g, while for the maximum 25% addition of bee pollen, the reducing capacity reached the value of 1323.8 μmol Fe(II)/100 g (Table 3) Juszczak et al. [28] also observed a rise in the reduction capacity from 303.81 to 582.62 μmol Fe(II)/100 g in samples of commercial honeys enriched with bee pollen. The reducing capacity of multiflower honey determined with the CUPRAC method was at a level of 77.81 μmol TE/100 g (Table 3). Similarly, as in the FRAP method, enrichment of honey with bee pollen resulted in a significant increase in the reducing capacity of honey. With 5% supplementation of honey with bee pollen, its reducing capacity increased to the level of 186.38 μmol TE/100 g, whereas for the maximum 25% addition of bee pollen, it reached the value of 449.04 μmol TE/100 g (Table 3). A proportional increase in the content of biologically active substances, in particular phenolic acids and flavonoids, caused by an addition of bee pollen (Table 1) resulted in an increase in antiradical and antioxidant activities, as well as in reducing capacity (Table 3). Consequently, the dependencies known from the literature data were observed [16,25]. The total phenolic content correlated significantly with the total antioxidant activity (r = 0.9936), antiradical activity against ABTS^•+^ (r = 0.9936), and DPPH^•^ (r = 0.9929) and with the reducing capacity evaluated using FRAP (r = 0.9835) and CUPRAC (r = 0.9901) methods. Similar high dependencies were observed for the total flavonoid content (r = 0.9937, 0.9931, 0.9910, 0.9885, and 0.9924) and the total phenolic acid content (r = 0.9677, 0.9721, 0.9589, 0.9647, and 0.9846), as well as the parameters characterizing antioxidant properties. Similar significant dependencies were established for the total anthocyanin and carotenoid contents and for individual flavonoid and phenolic acid contents.

### 3.4. The Impact of Bee Pollen on the Sensory Profile of Multiflower Honey

**Figure 1:**
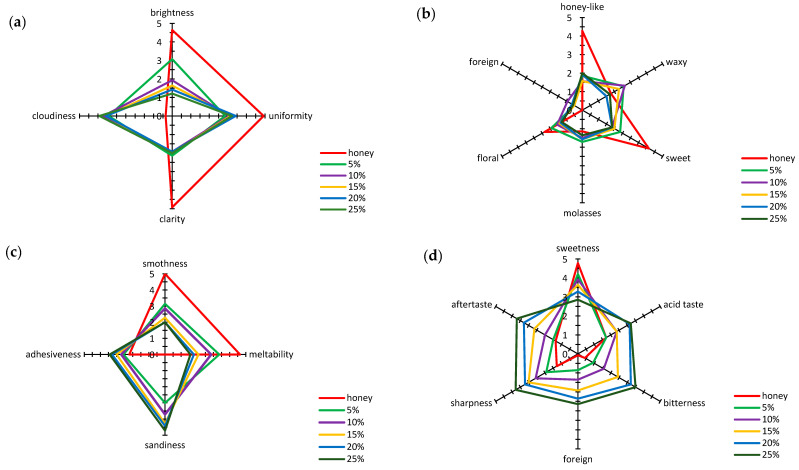
The results of sensory profiling analysis for the (**a**) color, (**b**) smell, (**c**) texture, and (**d**) taste of multiflower honey and honey samples enriched with bee pollen.

To analyze the impact of bee pollen addition on honey’s sensory characteristics, its color, smell, texture, and taste were evaluated. Figure 1a shows average results of the multiflower honey color evaluation and honey samples with increasing bee pollen content. The following sensory descriptors were analyzed to estimate the color: brightness, clarity, cloudiness, and uniformity. The mulitflower honey was estimated as very bright, clear, and highly uniform, with an average score ranging from 4.64 to 5.00. Moreover, the performed analysis did not show clouding. The increasing addition of bee pollen to honey reduced its brightness (Figure 1a). Already a 5% addition of bee pollen decreased the honey’s brightness score to a mean value of 3.07, and with 25% addition of bee pollen, the honey’s brightness dropped to a level of 1.21. The pollen addition also reduced the score for honey uniformity, clarity, and cloudiness. In the honey samples with bee pollen addition, the color uniformity scored 3 points on average, and differences between the scores for honey samples with different added amounts of bee pollen were slight.

The clarity of honey samples supplemented with bee pollen also scored between 2.14 to 1.93, and differences between mean scores for individual samples were insignificant. The observed reduced brightness, uniformity, and clarity of the honey samples with bee pollen addition led to a clearly visible increase in their cloudiness. The average score for this property of honey samples with bee pollen addition ranged from 3.43 to 3.93; however, the level of pollen addition itself had a minor influence on statistical variation in the average score.

The smell of the multiflower honey that was not enriched with bee pollen was described by evaluators as strongly noticeable, sweet, and honey-like (Figure 1b). The intensity of the floral smell impression was described by the evaluators as moderately noticeable. Both molasses and waxy smells in the multiflower honey were described by evaluators as barely perceptible, while a foreign smell itself was not noticeable. The increasing addition of bee pollen to honey led to a significant reduction in the intensity of the perceived honey-like and sweet smell. It was also observed that the presence of bee pollen only slightly influenced the noticing of molasses and floral smells and the intensity of the perceived waxy smell. Furthermore, the sensory panel did not observe a significant impact of bee pollen addition on the increased intensity of a foreign smell, and this indicates that the addition of bee pollen does not introduce a foreign smell, although it clearly modifies the natural smell of honey.

The next evaluated characteristic of the honeys was their texture (Figure 1c). The evaluators specified the level of intensity of their impression of adhesiveness, meltability, sandiness, and smoothness. The texture of the multiflower honey not enriched with bee pollen was characterized by strongly noticeable meltability and smoothness. This type of honey was described by the evaluators as moderately adhesive, while its sandiness was unnoticeable. The increasing addition of bee pollen significantly changed the intensity of the evaluated texture descriptors (Figure 1c). An increased intensity of sandiness was observed, which was very strongly noticeable for honey samples with 25% bee pollen addition, and an increase in adhesiveness, with an accompanying significant drop in smoothness and meltability, described by the evaluators at a level of moderate intensity of perception.

Tastiness represents the overall experienced taste, smell, and sensory impressions during product consumption. In the taste assessment conducted, the multiflower honey was assessed as very sweet with other characteristics, i.e., acid, bitter, and sharp tastes, slightly noticeable or noticeable at a threshold level, while perceptibility of a foreign taste intensity was assessed as unnoticeable (Figure 1d). The increasing addition of bee pollen significantly influenced the evaluated taste determinants (Figure 1d). A significant drop in perceived sweetness was found, down to moderate intensity with 25% bee pollen addition. The observed increase in the intensity of perceived acid, bitter, and sharp tastes was described as moderately noticeable in the evaluated samples of honey with the highest addition of bee pollen. An addition of bee pollen to honey also influenced the intensity of the perceived aftertaste, which increased to a moderate level, and of a noticeable foreign taste, from barely noticeable up to moderate, at 5% and 25% bee pollen addition, respectively.

**Figure 2:**
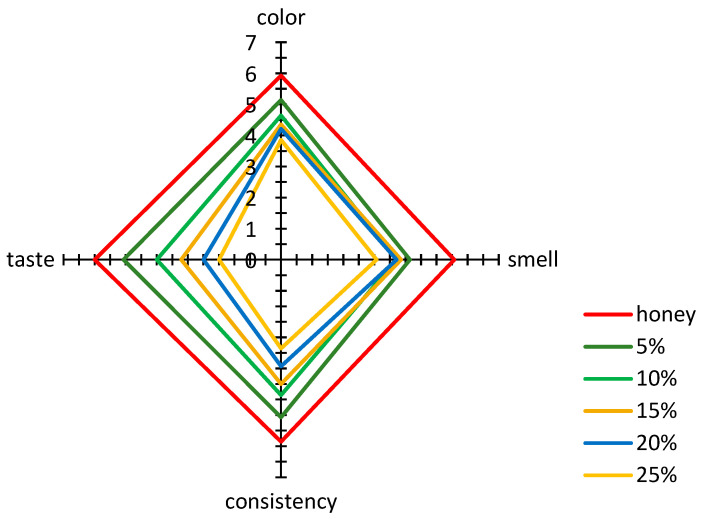
Results of the acceptability evaluation of multiflower honey and honey samples enriched with bee pollen.

The overall acceptance of basic distinguishing sensory parameters, such as color, smell, texture, and flavor with the addition of bee pollen, as evaluated according to the hedonic scale, is shown in Figure 2. The increasing addition of pollen to multiflower honey resulted in a decrease in the acceptability of the honey color due to a clear increase in its cloudiness and a decrease in clarity and uniformity (Figure 2).

A statistically significant correlation was observed between the results of color acceptability evaluation according to the hedonic scale and the results obtained by sensory profiling. The color acceptability was significantly positively correlated with brightness (r = 0.9849) and clarity (r = 0.8357) and negatively correlated with cloudiness (r = −0.8640). Similarly, as in the case of color, the enrichment of honey with bee pollen resulted in a drop in acceptance of its smell, resulting from the reduced intensity of perceived honey-like (r = 0.8631), sweet (r = 0.9518), and floral (r = 0.9378) smells. The evaluation of honey consistency using the sensory profiling method resulted in the finding that the addition of bee pollen significantly influenced the increased perception of unwanted sandiness, thus negatively affecting the perception of desirable characteristics, i.e., meltability and smoothness. The calculated values of the linear correlation coefficients indicated a significant positive correlation between acceptability and smoothness (r = 0.9222) and meltability (r = 0.9732) and a negative correlation with sandiness (r = −0.9050). The increasing addition of bee pollen also significantly influenced a decrease in the acceptability of flavor, associated with a reduced perception of sweetness and an increased perception of acid, bitter, and sharp tastes. The flavor acceptability was significantly positively correlated with noticeable sweetness (r = 0.9875), while it was negatively correlated with the perception of acid (r = −0.9516), bitter (r = −0.9905), foreign (r = −0.9973), and sharp (r = −0.9938) tastes and aftertaste (r = −0.9727).

### 3.5. The Impact of Bee Pollen Addition on the Honey Quality Parameters

Honey placed on the market must be of the required quality. In Polish legislation, the requirements for the commercial quality of honey are regulated by the Regulation of the Minister of Agriculture and Rural Development from 2003, as amended [36]. These regulations, in accordance with Council Directive (EC) 2001/110 [37], decide the threshold values for the physical and chemical parameters that are essential for honey quality. One of the parameters characterizing honey quality is its water-insoluble matter content, including bee bread and pollen grains, and propolis, as well as fragments of bees, algal and fungal cells, and bacterial spores. The total content of water-insoluble substances in multiflower honey and in honey samples supplemented with bee pollen is shown in Table 4. The multiflower honey under study contained 0.06 g of substances insoluble in water per 100 g, which meets the legislative requirements of 0.1 g/100 g specified in the Regulation of the Minister of Agriculture and Rural Development [36]. Honey enrichment with bee pollen significantly contributed to an increase in the level of insoluble matter. The addition of 5% bee pollen resulted in an increase in the content of these substances to 1.78 g/100 g. Each successive increase in the bee pollen content resulted in an increase in the insoluble matter content, reaching the level of 9.30 g/100 g for the maximum pollen addition of 25%. Juszczak et al. [38] observed that the addition of bee products to honey had a significant influence on the content of water-insoluble substances as a result of their poor solubility in water. The cited authors also report that enrichment of multiflower honey with bee products may lead to even a four times increase in the content of insoluble matter.

Honey acidity results from the content of organic acids and depends on the type of initial material and the bee species. Only small amounts of organic acids come from plants. The majority of them are produced from glucose by relevant enzymes originating from bee salivary glands. The determination of the free acidity of honey is useful in for evaluating its quality and for distinguishing between nectar and honeydew honeys. Immature honey is characterized by low acidity, and when the acceptable content of free organic acids in honey is exceeded, it may imply honey fermentation and/or excessive growth of microorganisms. The free acidity of multiflower honey and the honey samples enriched with bee pollen is shown in Table 4. For the multiflower honey, the value of this parameter was 22.87 mval/kg, and this conforms previous literature data reported for Polish multiflower honeys [38] and is consistent with the requirements of the Regulation of the Minister of Agriculture and Rural Development [36] specifying the acceptable value of free acidity as 50 mval/kg. The addition of bee pollen to honey resulted in a significant rise in the honey’s free acidity. Already at 5% addition of bee pollen, free acidity increased by ca. 100%, reaching a value of 45.33 mval/kg. Each successive increase in the bee pollen content led to a further proportional increase in the acidity, to a maximum value of 145.7 mval/kg for 25% addition of bee pollen. Bee pollen contains both organic acids as well as large amounts of amino acids, and this significantly influences the rise in honey acidity with the addition of this bee product. As Thakur and Nanda [1] reported, bee pollen acidity can reach even 290 to 400 mval/kg, depending on its geographical origin.

The specific conductivity of honey solution, as another quality parameter, depends on the honey composition, mainly on the content of its mineral ingredients and organic acids. The quality requirements for honey specify the maximum acceptable value of conductivity specific for nectar honeys at a level of 0.8 mS/cm [36]. The specific conductivity for the multiflower honey was established at a level of 0.50 mS/cm (Table 4), and this confirms that legal requirements are met, and the obtained value was within the range of values reported for Polish multiflower honeys [35,38]. A significant addition of bee pollen resulted in a proportional increase in the specific conductivity, amounting to 1.244 mS/cm for the maximum 25% of bee pollen. The increased content of mineral ingredients in bee pollen led to an increase in the conductivity. As Thakur and Nanda [1] reported, the ash content in bee pollen can reach 7.75%, depending on its geographic origin.

Carbohydrates found in honey represent the most numerous group of compounds, with simple reducing sugars prevailing. The total content of fructose and glucose in nectar honey cannot be lower than 60 g/100 g of honey [36]. The amount of glucose and fructose in the multiflower honey and in the honey samples supplemented with bee pollen is shown in Table 4. Regarding the investigated multiflower honey, the amount of glucose and fructose was determined at a level of 25.53 and 39.62 g/100 g, respectively. The total content of these sugars was 65.15 g/100 g, and this is consistent with the Regulation of the Minister of Agriculture and Rural Development [36] requirements. Bee pollen addition to multiflower honey resulted in a reduction in the content of reducing sugars (Table 4). At the highest bee pollen concentration, the glucose content dropped to 19.99 g/100 g and the fructose content decreased to 34.54 g/100 g. Kieliszek et al. [3] reported that bee pollen contains smaller quantities of simple sugars than multiflower honey does. Therefore, when part of the honey is replaced by bee pollen, the content of discussed saccharides in the product decreases, and with the highest bee pollen share, the total glucose and fructose content is below the minimum specified in relevant regulations [36]. The saccharose content is another quality requirement for honey, and it should not exceed 5 g/100 g. The analyzed multiflower honey met the requirements in that respect, as it contained 1.83 g of saccharose per 100 g. As bee pollen does not contain a significant amount of this sugar, its addition to honey is not of significant importance. A significant influence of honey supplementation with bee pollen on the decrease in the saccharose content was only observed when the bee pollen content exceeded 20%.

Another factor that determines the quality of natural honey is the presence of 5-hydroxymethylfurfural (HMF), and its increased content reveals that this product is stored under inappropriate conditions, in particular at an increased temperature. The increased HMF content may also be a result of honey overheating, e.g., to facilitate its filtration or to adulterate it by using inverted sugar obtained by acid hydrolysis. In accordance with the Regulation of the Minister of Agriculture and Rural Development [36], the maximum HMF content in honey cannot exceed 40 mg/kg. In the multiflower honey, the HMF content amounted to 10.76 mg/kg, and the bee pollen addition did not significantly affect the HMF content in enriched honey samples (data not shown). This confirms previous literature data concerning samples of commercial honeys supplemented with bee products [38]. The proline content in the multiflower honey and in honey samples enriched with bee pollen is shown in Table 4. The analyzed multiflower honey contained 32 mg of proline per 100 g. With the increasing addition of bee pollen, a proportional increase in the proline content was observed, up to 106 mg/100 g of honey for 25% addition of bee pollen.

## 4. Conclusions

The present study investigated the impact of multiflower honey enrichment with micronized bee pollen on the contents of some biologically active substances, antioxidant activity, and qualitative and sensory properties. The obtained results indicated that the addition of micronized bee pollen to multiflower honey led to a significant increase in the polyphenolic content, including phenolic acids and flavonoids, of which gallic acid and kaempferol were present in the highest amounts. Moreover, the addition of bee pollen to honey and an increase in the content of phenolics resulted in an increase in antiradical, antioxidant, and reducing activities. In contrast, honey enrichment with bee pollen led to its deterioration in terms of sensory characteristics. For color, a decrease in brightness, clarity, and uniformity was observed, accompanied by an increase in cloudiness. Regarding the smell characteristics, a significant reduction in the perception of honey-like and sweet tastes was found. In the evaluation of texture, an increased intensity of sandiness was observed, which was very strongly noticeable for honey samples with the highest bee pollen addition, and an increase in adhesiveness, with an accompanying significant drop in smoothness and meltability. The evaluation of taste indicated a visible decrease in noticed sweetness and an increase in the intensity of acid, bitter, and sharp tastes and of an aftertaste. The introduction of bee pollen to honey led to an increase in free acidity, the content of insoluble matter, specific conductivity, and proline content, accompanied by the observed reduction in glucose and fructose content. The results obtained in the present study indicate that honeys supplemented with bee pollen can be an excellent and valuable source of natural antioxidants; however, the use of bee pollen as an additive strongly depends on the changes in sensory properties and the acceptance of consumers.

## Figures and Tables

**Table 1:** The total phenolic, flavonoid, phenolic acid, anthocyanin, and carotenoid content in multiflower honey and honeys enriched with bee pollen.

Addition of Bee Pollen (%)	Total Phenolic Content (mg GAE/100 g)	Total Flavonoid Content (mg QE/100 g)	Total Phenolic Acid Content (mg CAE/100 g)	Total Anthocyanin Content (mg/100 g)	Total Carotenoid Content (mg/100 g)
0	30.75 ± 0.25	2.77 ± 0.29	11.02 ± 0.68	2.01 ± 0.05	0.138 ± 0.001
5	63.33 ± 0.27	5.94 ± 0.25	16.65 ± 0.19	4.02 ± 0.05	0.311 ± 0.004
10	89.42 ± 0.61	8.38 ± 0.19	17.08 ± 0.23	5.57 ± 0.38	0.934 ± 0.001
15	136.63 ± 0.44	12.11 ± 0.48	20.32 ± 0.52	7.60 ± 0.19	1.404 ± 0.002
20	156.13 ± 0.92	14.25 ± 0.27	21.26 ± 0.39	9.16 ± 0.09	1.726 ± 0.001
25	178.26 ± 1.13	16.39 ± 0.16	24.44 ± 0.17	11.32 ± 0.10	2.333 ± 0.001
LSD_0.05_	0.83	0.36	0.51	0.22	0.003

**Table 2:** The content of flavonoids and phenolic acids (mg/100 g) in multiflower honey and honeys enriched with bee pollen.

Component	Bee Pollen Addition (%)						LSD_0.05_
	0	5	10	15	20	25	
Chrysin	0.014 ± 0.001	0.015 ± 0.001	0.020 ± 0.001	0.037 ± 0.001	0.046 ± 0.001	0.054 ± 0.002	0.002
Galangin	0.023 ± 0.001	0.045 ± 0.002	0.073 ± 0.001	0.109 ± 0.007	0.130 ± 0.009	0.170 ± 0.011	0.008
Kaempferol	0.049 ± 0.004	0.392 ± 0.019	0.664 ± 0.042	1.478 ± 0.023	1.867 ± 0.066	2.183 ± 0.041	0.047
Quercetin	0.040 ± 0.001	0.237 ± 0.011	0.296 ± 0.005	0.392 ± 0.011	0.642 ± 0.007	0.731 ± 0.016	0.012
Ferulic acid	0.095 ± 0.005	0.191 ± 0.005	0.260 ± 0.006	0.296 ± 0.004	0.333 ± 0.010	0.503 ± 0.009	0.009
Gallic acid	0.217 ± 0.004	0.526 ± 0.019	0.756 ± 0.016	1.115 ± 0.035	1.339 ± 0.027	1.494 ± 0.071	0.045
*p*-Hydroxybenzoic acid	0.040 ± 0.003	0.068 ± 0.001	0.073 ± 0.002	0.078 ± 0.002	0.088 ± 0.001	0.133 ± 0.001	0.002
Caffeic acid	0.026 ± 0.000	0.034 ± 0.001	0.039 ± 0.000	0.042 ± 0.002	0.071 ± 0.004	0.163 ± 0.011	0.006
*p*-Coumaric acid	0.136 ± 0.006	0.296 ± 0.002	0.340 ± 0.003	0.395 ± 0.015	0.441 ± 0.011	0.485 ± 0.018	0.014
Protocatechuic acid	0.070 ± 0.003	0.167 ± 0.002	0.182 ± 0.009	0.254 ± 0.011	0.363 ± 0.003	0.535 ± 0.020	0.013

**Table 3:** The antioxidant, antiradical, and reducing activities of multiflower honey and honey samples enriched with bee pollen.

Bee Pollen Addition (%)	Antioxidant Activity (mmol AAE/100 g)	ABTS^•+^ (mmol TE/100 g)	DPPH^•^ (mmol TE/100 g)	FRAP (µmol Fe(II)/100 g)	CUPRAC (µmol TE/100 g)
0	9.24 ± 0.12	1.78 ± 0.02	0.26 ± 0.00	233.9 ± 0.7	77.81 ± 1.44
5	9.90 ± 0.14	5.40 ± 0.02	0.71 ± 0.01	497.0 ± 0.6	186.38 ± 4.09
10	10.18 ± 0.18	7.38 ± 0.10	1.25 ± 0.01	681.7 ± 1.2	266.59 ± 3.48
15	11.25 ± 0.25	10.95 ± 0.09	1.85 ± 0.02	831.9 ± 0.5	343.86 ± 2.05
20	11.81 ± 0.23	12.88 ± 0.19	2.05 ± 0.01	1163.8 ± 3.4	375.62 ± 0.96
25	12.41 ± 0.31	13.48 ± 0.20	2.22 ± 0.01	1323.8 ± 1.1	449.04 ± 4.72
LSD_0.05_	0.37	0.16	0.01	2.01	3.89

**Table 4:** The qualitative parameters of multiflower honey and honey samples enriched with bee pollen.

Bee Pollen Addition (%)	Insoluble Matter (g/100 g)	Free Acidity (mval/kg)	Specific Conductivity (mS/cm^3^)	Glucose Content (g/100 g)	Fructose Content (g/100 g)	Proline Content (mg/100 g)
0	0.06 ± 0.01	22.87 ± 0.15	0.500 ± 0.001	25.53 ± 0.51	39.62 ± 0.91	32.35 ± 0.43
5	1.78 ± 0.02	45.33 ± 1.14	0.652 ± 0.000	25.25 ± 0.51	38.71 ± 0.89	55.12 ± 0.11
10	3.65 ± 0.02	70.40 ± 0.40	0.822 ± 0.001	23.51 ± 0.06	35.97 ± 0.24	77.57 ± 0.23
15	5.98 ± 0.05	95.80 ± 1.37	0.964 ± 0.000	23.26 ± 0.26	35.35 ± 0.26	91.98 ± 0.54
20	7.39 ± 0.16	119.23 ± 1.76	1.115 ± 0.000	23.00 ± 0.24	35.32 ± 0.16	45.90 ± 0.93
25	9.30 ± 0.03	145.70 ± 0.60	1.244 ± 0.000	19.99 ± 0.68	34.54 ± 0.18	106.52 ± 1.21
LSD_0.05_	0.05	1.22	0.002	0.54	0.68	1.94

## Data Availability

All data concerning the presented studies are included in the paper.
